# A compact tri-notched flexible UWB antenna based on an inkjet-printable and plasma-activated silver nano ink

**DOI:** 10.1038/s41598-024-62253-2

**Published:** 2024-05-18

**Authors:** Wendong Yang, Xun Zhao, Zihao Guo, Haoqiang Sun, Emil J. W. List-Kratochvil

**Affiliations:** 1 School of Electronic and Information Engineering, Liaoning Technical University, Huludao City, 125105 China; 2 Institut für Physik, Institut für Chemie, IRIS Adlershof, Humboldt-Universität zu Berlin, 12489 Berlin, Germany; 3 Helmholtz-Zentrum Berlin für Materialien und Energie GmbH, 14109 Berlin, Germany

**Keywords:** Flexible electronics, Antennas, Conductive inks, Triple notch, Plasma sintering, Electrical and electronic engineering, Materials for devices

## Abstract

The rapid development of ultrawideband (UWB) communication systems has resulted in increasing performance requirements for the antenna system. In addition to a wide bandwidth, fast propagation rates and compact dimensions, flexibility, wearability or portability are also desirable for UWB antennas, as are excellent notch characteristics. Although progress has been made in the development of flexible/wearable antennas desired notch properties are still rather limited. Moreover, most presently available flexible UWB antennas are fabricated using environmentally not attractive subtractive etching-based processes. The usage of facile additive sustainably inkjet printing processes also utilizing low temperature plasma-activated conductive inks is rarely reported. In addition, the currently used tri-notched flexible UWB antenna designs have a relatively large footprint, which poses difficulties when integrated into miniaturized and compact communication devices. In this work, a silver nano ink is used to fabricate the antenna via inkjet printing and an efficient plasma sintering procedure. For the targeted UWB applications miniaturized tri-notched flexible antenna is realized on a flexible polyethylene terephthalate (PET) substrate with a compact size of 17.6 mm × 16 mm × 0.12 mm. The antenna operates in the UWB frequency band (2.9–10.61 GHz), and can shield interferences from WiMAX (3.3–3.6 GHz), WLAN (5.150–5.825 GHz) and X-uplink (7.9–8.4 GHz) bands, as well as exhibits a certain of bendability. Three nested "C" slots of different sizes were adopted to achieve notch features. The simulation and test results demonstrate that the proposed antenna can generate signal radiation in the desired UWB frequency band while retaining the desired notch properties and having acceptable SAR values on-body, making it a viable candidate for usage in flexible or wearable communication transmission devices. The research provides a facile and highly efficient method for fabricating flexible/wearable UWB antennas, that is, the effective combination of inkjet printing processing, flexible substrates, low temperature-activated conductive ink and antenna structure design.

## Introduction

Ultrawideband (UWB) wireless communication systems, with frequencies ranging from 3.1 to 10.6 GHz, Ref.^1^ have received a lot of interest due to their attractive features of high data rate, low power consumption, accurate positioning and large capacity, Refs.^2–5^ which show application potential in IoT^6^, radar systems^7^, wireless body area network (WBAN) ^8^ and medical^9^. With the rapid development of wireless communication technology, high performance UWB antennas are required by UWB systems. Apart from the need for wide bandwidth, fast data rates and small dimensions, flexibility, wearability or portability are also desirable for the UWB antenna. On the other hand, while UWB antennas have a highly reliable quality, signals from several narrowband systems, such as world interoperability for microwave access (WiMAX) system, wireless local area networks (WLAN) system, and X-band^4,10^, may cause interferences to UWB systems and affect its communication quality, which need to be filtered out to improve the quality of UWB systems. Hence, flexible/wearable antennas with notch properties for UWB communication systems have received much attention.

Up to now, much effort has been made in the development of flexible/wearable antennas with notch properties, and a variety of notch techniques have been adopted, such as etching various shaped slot on the radiating patch^11–17^ or ground plane incorporation of electric ring resonator (ERR)^18^, split ring resonators (SRRs)^11,19–22^ or complementary split ring resonators (CSRRs)^23–26^, using parasitic elements^27,28^, defected ground structures (DGS) ^29–31^ and EBG resonators^32–35^, etc. For instance, Geyikoglu et al.^29^ demonstrated the design and implementation of a flexible UWB antenna with dual-band rejection capabilities for wearable biomedical devices. The antenna had a CPW fed circular and triangle structure, which was fabricated on Kapton a polyimide-based flexible substrate using air-brush-printing of a silver ink. The notch properties were realized by using two triangular-shaped spiral slots defected ground structures. Lakrit et al.^11^ presented a flexible UWB antenna with band notched characteristics for high-speed WLAN applications. The flexibility of the designed antenna was realized by using a flexible Teflon substrate. The antenna was 42.5mm × 30mm × 0.6mm in size and consisted of a slot loaded octagonal star-shaped patch with a partial ground plane for wide operating bandwidth. The proposed antenna is suitable for mounting on all types of surface area with band rejection characteristics. Ma et al.^16^ developed a flexible wearable UWB antenna with a notching function for wireless personal area network (WPAN) applications. The antenna was W shaped with dimensions of 40 mm ×30 mm ×1.5 mm. It was printed on a Neoprene substrate and fed with a 50ohm microstrip. Abutarboush et al.^30^ proposed a flexible, wideband, and screen-printed antenna. The antenna has the form of a coplanar-waveguide, with a total size of 55 mm × 40 mm × 0.125 mm, and was composed of two inverted L-shaped elements, a matching stub and a defected ground structure (DGS). It achieved a bandwidth of 1.77–6.95 GHz and was suited for different wideband communication systems, notably functioning in the sub 7 GHz bands, such as the 5G applications. Roy et al.^36^ proposed a jeans-based narrow-band, flexible, wearable UWB antenna. The antenna had a total size of 38 mm × 23 mm × 0.6 mm and made up of two double-step rectangular sections connected by a microstrip line, allowing it to provide ultra-wideband coverage with notch characteristics over telemetry/mobile communication (4.4–4.99 GHz) and WLAN (5.15–5.85 GHz) bands. The antenna was tested for on-body use and yielded good results, making it ideal for wearable applications.

Although advances have been made in the development of flexible or wearable UWB antennas with notch characteristics, there are still some limitations. Firstly, most existing flexible UWB antennas with notch properties are fabricated using non-environmentally friendly subtractive etching methods. This approach consumes a large amount of metal materials, and has to go through a series of complicated processing steps, which results in a lot of waste liquid^37^. Secondly, the current developed antennas are relatively large in size, with lengths and widths greater than 30mm, which may pose difficulties in integrating them into miniaturized and compact communication devices, limiting their applications. Lastly, inkjet printing, a low-cost, scalable, environmentally friendly approach, has up to now rarely been utilized for the fabrication of flexible UWB antennas. This method directly deposits solution-processable materials onto the specific region of the substrate^38^, which shows enormous potential in the fabrication of flexible and wearable antennas since it does not require a mask and allows designs to be modified and printed at any time^39^. In addition, it also results in high-resolution patterns and compact designs. Therefore, exploiting a minimal, inkjet-printed, flexible UWB antenna with notch properties will be interesting.

Conductive inks are essential for flexible UWB antennas since they are used to create the conductive part of the antenna. Metals such as gold, silver or copper have excellent conductivity, so conductive inks based on these materials have been rapidly developed^40–48^. However, low-temperature metallization is the one of critical issues that need to be addressed with such inks in order to achieve desirable electrical performance. This is due to the fact that the majority of flexible substrate materials on which the ink is printed, such as polymer films and paper, are temperature sensitive. To fabricate antennas on flexible substrates, it is crucial that the whole processing temperature should not damage the substrate and cause their deformation. Considering this, non-thermal post-processing procedures are preferred. Plasma sintering provides an efficient way for the rapid and low-temperature fabrication of metal patterns that utilizes a plasma flow to get rid of the capping agent protecting the nanoparticles, allowing them to be sintered to form electrically conductive structures^49–52^. By choosing hydrogen, oxygen, and nitrogen, the plasma can be reducing, oxidizing, and inert respectively. The temperature during plasma irradiation does not exceed 70 °C, which enables the use of low glass transition temperature flexible substrates^50^. To our knowledge, plasma-activated metal inks are presently mainly employed for optoelectronic device applications^50,52–56^, with just a few being used in antennas, and only in RFID antennas^57^. The reason may be that it requires innovative and integration of multiple disciplines such as material science, process technology and electromagnetic designs. Thus, it will be very meaningful to explore the application potential of plasma-activated metal inks in the fabrication of inkjet-printed-based flexible and wearable antennas with notch properties.

In this paper, the use of a plasma-activated silver nano ink for the fabricating a miniaturized tri-notched flexible antenna for UWB applications is presented. The ink, composed of monodisperse silver nanoparticles and alcohols, can be easily printed and produces favorable conductive patterns with a highly efficient plasma sintering. The proposed antenna has a compact size of 17.6 × 16 × 0.12 mm^3^. It covers the 2.9–10.61 GHz frequency range for UWB systems and achieves the desired notch characteristics even after different degrees of bending. Three nested "C" slots of different sizes are introduced on the radiating patch to achieve notch characteristics at the WiMAX, WLAN, and X uplink bands. An antenna prototype was created on a flexible PET substrate via inkjet printing, which results in signal radiation and the desired notch properties in the targeted UWB frequency band, making the antenna a good candidate for usage in flexible or wearable communication devices. The research is expected to provide guidance for the development of flexible/wearable antennas using low cost, environmentally friendly and highly efficient manufacturing processes.

## Antenna design

### Antenna configuration

The proposed flexible triple-notch antenna is designed to operate in the UWB frequency band of 3.1–10.6 GHz, which can shield interferences from WiMAX (3.3–3.6 GHz), WLAN (5.150–5.825 GHz) and X-uplink (7.9–8.4 GHz) bands.

**Figure 1:**
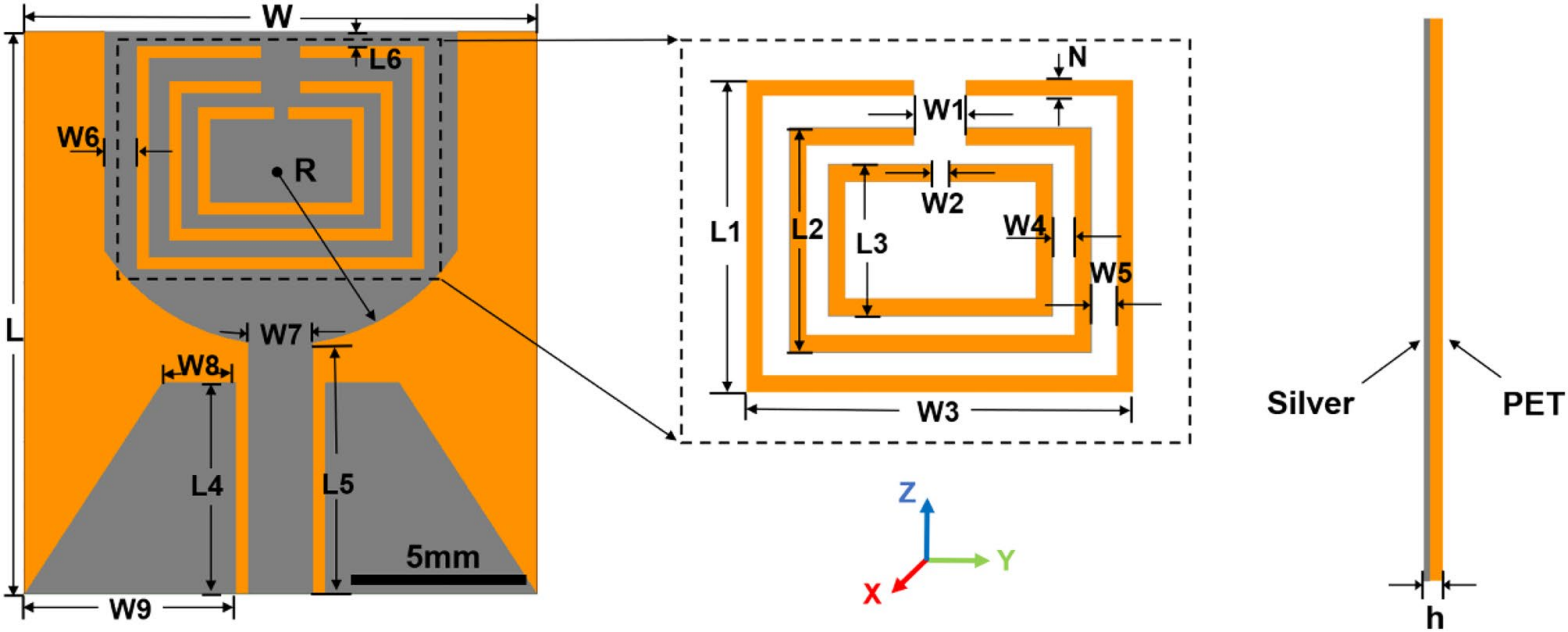
The geometrical shape of the proposed antenna.

Figure 1 depicts the geometrical shape of the proposed antenna. The orange part is the flexible PET substrate, while the grey part is the antenna pattern obtained by inkjet printing of commercial silver conductive ink. PET was chosen as the substrate because it has good flexibility and bendability. The permittivity (ε_r_), dielectric loss tangent (tanδ), and thickness of PET were 4, 0.01, and 0.12 mm, respectively.

The total dimensions of the antenna are 17.6 × 16 × 0.12 mm^3^, and it is a variant of the circular monopole antenna. It was made up of a ground plate, a feeder and a radiation patch with three "C"-shaped slots of different sizes. Selecting such antenna design has three advantages: (1) it is simple in structure and does not need any additional components, which reduces the complexity of the antenna; (2) the radiating patch has a smaller shape and a relatively smooth feedline transition. Such shape helps to reduce the size of the antenna while extending its bandwidth; (3) using a trapezoidal coplanar waveguide feed structure (CPW) can improve the antenna’s anti-interference performance and impedance matching while broadening its bandwidth. Besides, such a feeding structure is beneficial for inkjet printing; (4) the adoption of three nested "C"-shaped slots in the radiation patch not only helps to reduce the size of the antenna but also facilitates printing of the antenna prototype.

### Design processes

**Figure 2:**
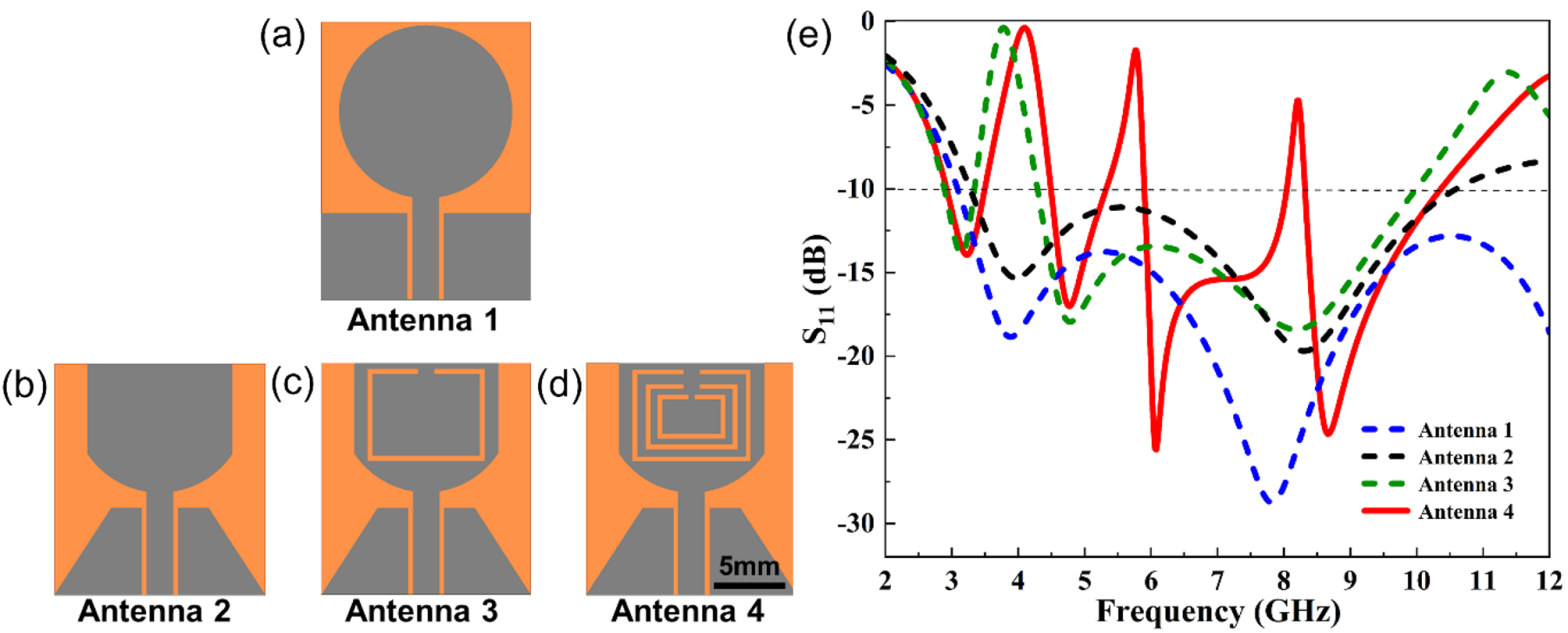
Design evolution processes of the proposed tri-notched flexible UWB antenna: (**a**–**d**) structures of Antenna 1–4, and (**e**) their corresponding S11 values.

The design went through four stages of structural evolution before reaching its ultimate structure, as illustrated in Fig.2. Firstly, Antenna 1, a circular monopole UWB antenna feed by a CPW structure was developed with dimensions of 21 × 16 × 0.12 mm^3^ (Fig. 2a). Then, Antenna 2 was obtained by cutting the top, side circular part and ground plate part of Antenna 1 separately. This was done to reduce the area of the radiation patch, ground plate and substate, achieving a compact monopole UWB antenna (Fig. 2b). Following that, a C-shaped slot was introduced on the radiating patch of Antenna 2 to create the structure of Antenna 3 and isolate the interference from the WiMAX band (Fig. 2c). Finally, two "C"-shaped slots were successively introduced on the radiating patch of Antenna 3 to filter out the interferences from WLAN and X uplink bands (Fig. 2d), resulting in the ultimate structure (Antenna 4). For parametric analysis, a 3D electromagnetic simulation software (ANSYS HFSS, Ansys Electronics 2023 R2) is employed.

### Notch principle

Here, the slotting technique was utilized to realize the notch^58^. Slotting is etching slots of different shapes on the ground plate, radiation unit and feeder line of the antenna. According to reference^58^, a reverse current and a reverse electric field can be created at the slotting position so that the antenna cannot radiate energy outward, thereby resulting in a notch. By etching the slots in the antenna structure, the current path can be blocked or changed to a certain extent to affect the current distribution on the antenna surface and achieve the notch purpose. The center frequency of the notch frequency band can usually be adjusted by changing the size and shape of the slot.

Based on the relationship between the center frequency of the notch frequency band and the length L of the slot (Eqs. (1) and (2)), Refs.^24,59^ three “C"-shaped slots were successively introduced on the radiating patch of the Antenna 2 to achieve notch properties. The outermost "C"-shaped slot was inserted to reject the 3.3–3.6 GHz WiMAX band, the middle "C"-shaped slot was introduced for 5.150–5.825GHz WLAN band and the inner "C"-shaped slot was used to reject the 7.9–8.4GHz bands of X-uplink, respectively.

1 $$L_{notch} { = }\frac{c}{{2f_{notch} \sqrt {\varepsilon_{eff} } }}$$

2 $$\varepsilon_{eff} \approx \frac{{\varepsilon_{r} + 1}}{2}$$

Where *L*_*notch*_ is the inner circumference of the slot; *c* is the speed of light; ƒ_notch_ is the notch center frequency; *ε*_*r*_ is the relative permittivity of the substrate; *ε*_*eff*_ is the effective dielectric constant.

Here, the position of the "C" slots needs to be paid to ensure that the currents of the "C" slots introduced later do not cancel out the current of the "C"-shaped slot introduced earlier. Considering the size of the radiation patch, we chose a smaller opening to increase the inner perimeter of the "C"-shaped slot, so increasing the notch center frequency and ultimately covering the desired notch frequency band. In addition, the opening orientations of the three "C"-shaped slots were made constant and set in a nested way in order to adjust the operating frequency band of the antenna and realize three notch bands.

Using Eqs. (1) and (2), the length of the slot, *L*, was calculated to be 23.1mm, 16.6mm and 11.7mm when the center frequencies ƒ_notch_ of the three notches were set to 4.1 GHz, 5.7 GHz and 8.1 GHz, respectively. After parameter optimization, the inner circumference of the outermost "C"-shaped slot, the middle "C"-shaped slot and the inner "C"-shaped slot were 27.6 mm, 19.6 mm and 13.6 mm, separately. And the total size of the antenna was measured to be 17.6 × 16 × 0.12 mm^3^, showing a 16.9% decrease in size over the initial circular monopole UWB antenna.

The S11 values of four antennas are shown in Figure 2e. Obviously, the Antenna 4 successfully achieved notch characteristics in the frequency bands of 3.5-4.45GHz, 5.5-6.4GHz, and 8.1-8.3GHz, with S11 less than -10dB in other UWB regions.

## Antenna prototype fabrication

### Materials and methods

The proposed flexible antenna was fabricated utilizing a commercial silver nano ink, BroadCON-INK550 (BroadTeko Co., Ltd., China). The ink has a viscosity of 8 cP and a surface tension of 29.3 dyne/cm, and contains 25–30 wt% silver nanoparticles. A PeJet-DP500 microelectronics printer equipped with piezoelectric printheads (BroadTeko Co., Ltd., China) was used for the printing of the ink. The minimum printed feature line width the printer can produce is ranging between 100 and 200μm, depending on the surface properties and the resulting spread of the ink of the substrate. The maximum printing area is 200 mm* 200 mm. The single drop volume used was 3 pl. During the experiments, the sample films and the designed antenna pattern were printed at a nominal resolution of 1440 dpi in unidirectional mode. Thermal or plasma sintering was used to treat the printed samples. Thermal sintering was performed in an oven for 60 min at temperatures of 70 °C, 90 °C, 110 °C, 130 °C and 150 °C. The plasma sintering was carried out for 30 min using a low-pressure argon plasma equipment (CSCPIA5, Shanghai Zhongbin Technology Co., Ltd., China) with a power value of 300 W.

### Characterization

The surface tension and contact angle of the silver nano ink were measured using a drop-shaped analyzer (Shengte, ST3900, Suchow, China). The size and morphology of silver particles in the ink were examined using a transmission electron microscope (TEM, FEI TecnaiTM G2 F-20 S-TWIN). The sample for TEM test was prepared by dripping the ethanol-dispersed silver nano ink over carbon-copper grids. The phases of the sintered films were identified using an X-ray diffraction (XRD, Rigaku) with Cu K*a* radiation and k = 0.15418 nm. A scanning electron microscope (SEM, ZEISS Gemini 300) and a surface energy disperse spectrometer (EDS, Oxford Xplore30) were used to examine the surface morphology and chemical composition of the sintered films. The thickness of the sintered films was measured using an optical profilometer (Bruker Contour GT-K, Germany), and the resistivity was determined using a four-point probe analyzer. The S11 values of the antenna prototype was measured with a vector network analyzer (Keysight E5063A). A cyclic bending testing method was used to evaluate the bendability of the printed flexible antenna.

## Results and discussion

### Plasma-activated printable silver nano ink

For decades printable metal inks with high conductivity and operational stability have been successfully utilized in the fabrication of conductive patterns^60–63^. Silver nanoparticles possess high electrical conductivity and low melting point (caused by thermodynamic size effect^64^), making them suitable for ink applications. Silver-based conductive inks are less expensive than platinum and gold-based metal inks, and they are more stable than copper-based inks and less prone to oxidation. Thus, a commercial silver nano ink was chosen here for the designed antenna.

**Figure 3:**
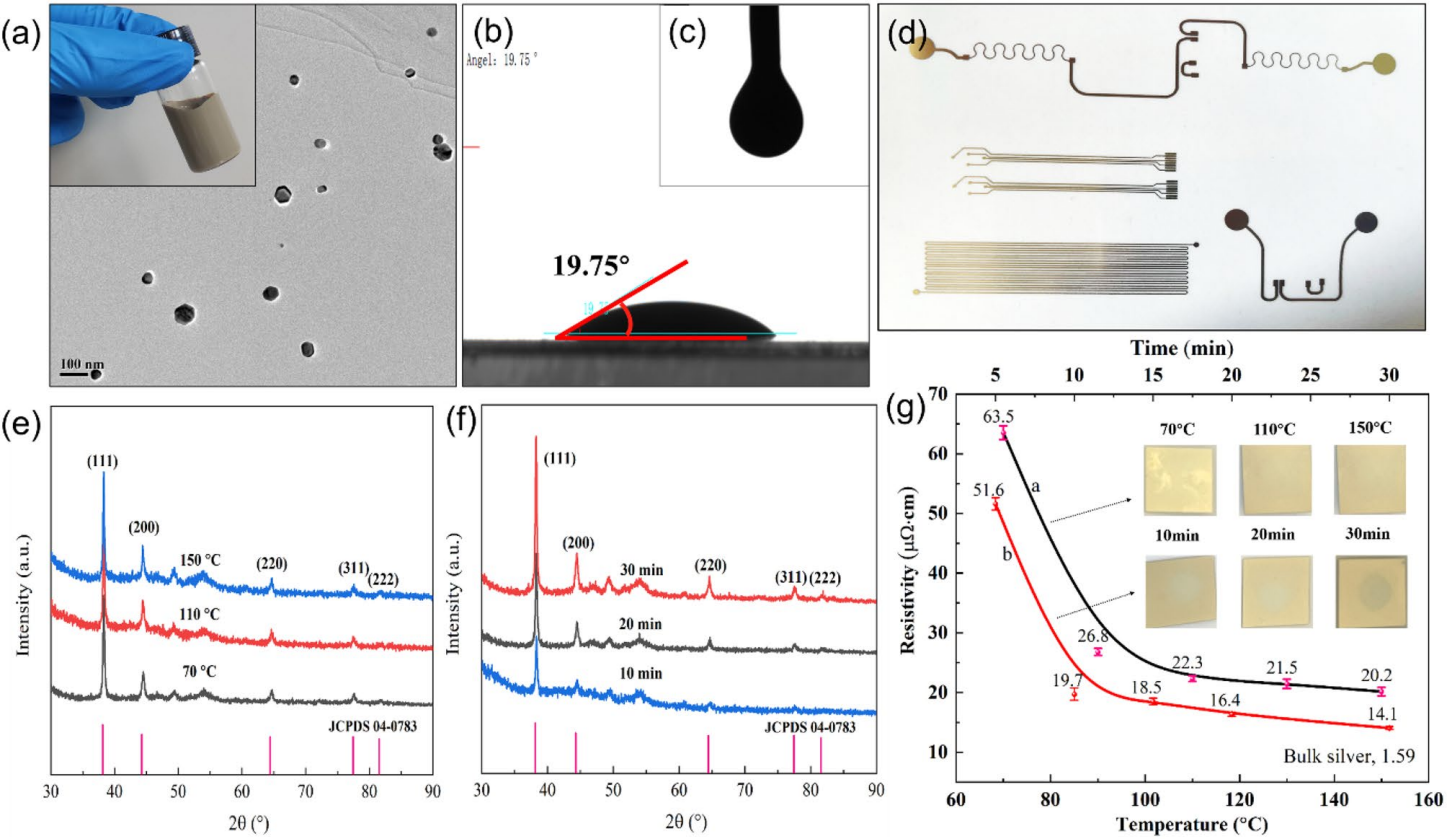
(**a**–**c**) TEM image, contact angle and surface tension of the silver nano ink; (**d**) the printed conductive patterns; (**e**,**f**) XRD patterns of the printed silver films with thermal sintering at 70, 110 and150°C and plasma sintering at 300W for 10, 20 and30 min; and (**g**) Resistivity values of the silver films against temperature or plasma time

The morphology and size of the metallic particles in the silver ink were observed by TEM firstly. As shown in Fig. 3a, the silver particles in ink exhibit spherical or hexagonal morphologies, with sizes smaller than 30 nm, which is advantageous for the electrical property. This is because a conjunction of different shapes and sizes provides multiply contact points and improved packaging efficiency. In addition, it is beneficial to the inkjet printing since it can avoid clogging and blocking of print-head nozzles.

For printing, the fluid properties of the ink are vital because they impact printing quality, which in turn influences the electrical and mechanical properties of the printed patterns. For antennas, their radiation performance is usually related to three parameters: effective length, effective area and impedance. The effective length characterizes the antenna’s efficiency in transmitting and receiving electromagnetic waves, while the effective area indicates the antenna’s capacity to capture and focus electromagnetic waves^65^. Impedance is the sum of resistance and reactance, which is governed by the ink used. During printing, if there are significant geometric differences between the printed and the designed antenna, the values of these three parameters will inevitably change, which will in turn cause changes in the antenna’s radiation performance. Therefore, the printing quality of the antenna pattern is crucial. In other words, the fluid properties of the ink are important. To jet smoothly, the rheological parameters of the ink, such as viscosity, surface tension and wettability, must be carefully evaluated. Favorable wettability is a prerequisite to ensure good adhesion between the ink and flexible substrates, while surface tension is useful in determining whether an ink will remain where it is deposited and how wide it will be after drying^61^. Both are critical for obtaining well-defined, high-resolution and conductive antenna patterns. Based on the Ref.^60^, in the case of the used piezoelectric print head, the ink viscosity should be in the range of 8-15 cP, while the surface tension should be in the range of 25–35 dyne/cm. As seen in Fig. 3b and c, the ink has a surface tension of 29.1 dyne/cm and a contact angle of 19.75° is measured on the PET substrate. The viscosity is measured to be 8.2 cP. These values are within the range required for piezoelectric print heads, suggesting that the ink is suitable for inkjet-printing. As expected, silver conductive patterns in different shapes were successfully fabricated (Fig. 3d) on flexible PET substrates, proving the good printability and applicability of the ink.

Metal nano inks, in general, do not have intrinsic conductivity and require additional post-processing to remove the solvent, stabilizing agents from the surface of the nanoparticle and induce a coalescence of the nanoparticles in order to achieve good conductivity. Together with drying and curing, sintering processes are considered post treatments. Once this process takes place a continuous percolating network is formed throughout the printed features, resulting in electrical property. Three dominant factors are mainly responsible for the electrical performance of the metal ink films after sintering: the sizes of the particles produced, the degree of organic residues and the film’s densification. This is the result of both the conductive channel effect and the tunneling effect, as proved by previous work^66–69^.

Here, thermal sintering and plasma sintering were adopted for the printed square films in order to identify a more efficient way. XRD, SEM, EDS and four-probe analyses were employed to investigate the physical phase, morphology and resistivity of the films produced by both sintering approaches.

Figure 3e and 3f show the crystalline structure of thermal and plasma sintered films, respectively. All samples exhibit peaks corresponded to (111), (200), (220), (311), and (222) planes of a face-centered cubic silver crystal, respectively, revealing the formation of metal silver films. The intensity of diffraction peaks of silver increases with the increase of sintering temperature or time, indicating an improved crystallinity. The resistivity of the sintered silver films against temperature or plasma time is given in Fig. 3g. A significant decrease in resistivity was found as the temperature or time increases, from 63.5 μΩ·cm of 70 °C to 20.2 μΩ·cm of 150 °C for thermal sintering and from 51.6 μΩ·cm of 5 min to 14.1 μΩ·cm of 30 min for plasma sintering. In comparison with thermal sintering, plasma sintering takes less time to achieve similar electrical performance, as proved by the resistivities obtained using thermal sintering at 150 °C for 60 min (20.2 μΩ·cm) and plasma sintering at 300 W for 10 minutes (19.7 μΩ·cm). The decrease in resistivity with the temperature or time can be easily understood because silver nanoparticles are better connected, and the solvents and organic layer evaporate or/and decompose adequately. The resistance values measured at various points on the film are basically the same, meaning that the printed film has homogeneous electrical properties.

**Figure 4:**
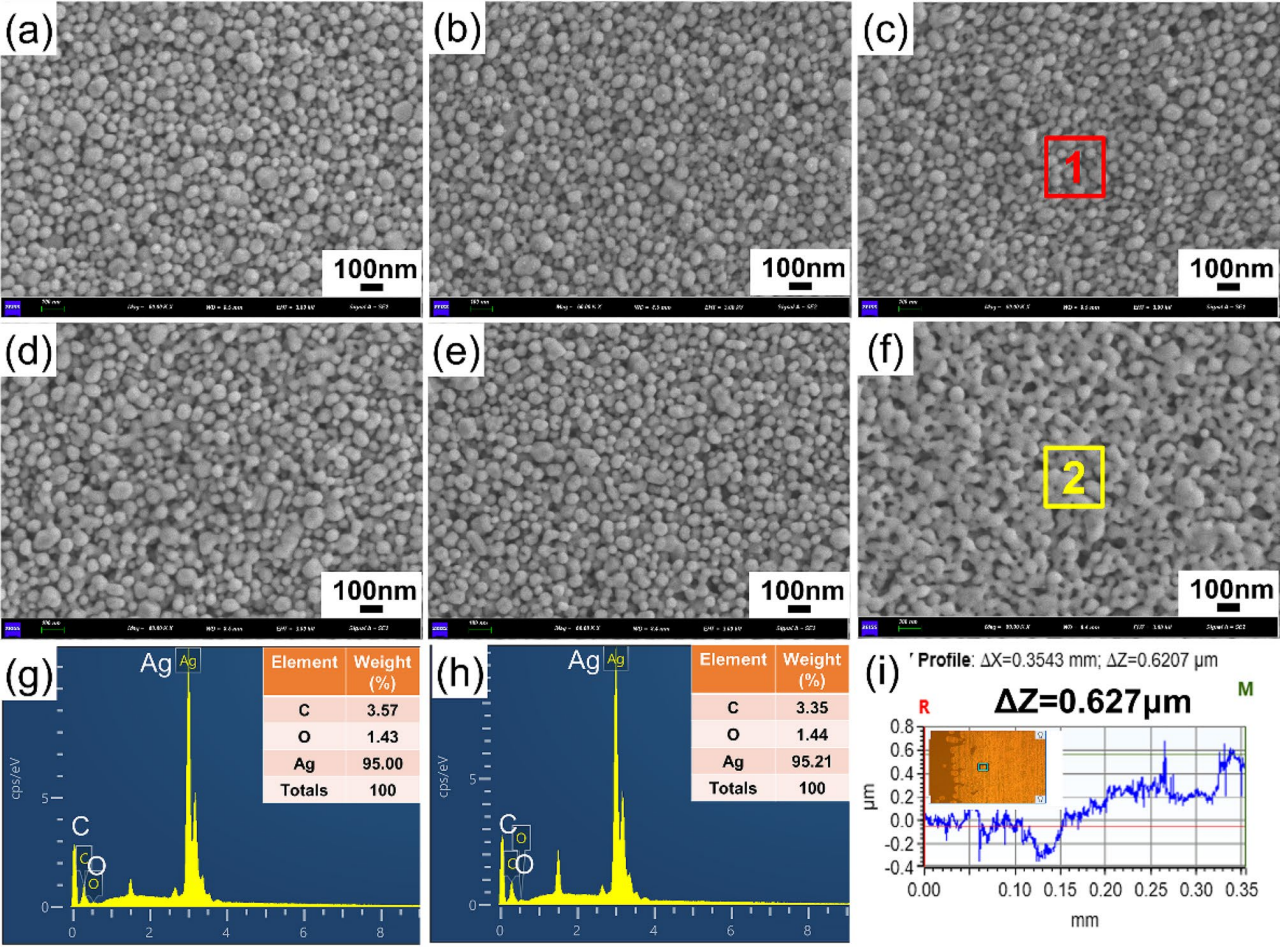
Surface morphologies and EDS results of the printed silver films with thermal sintering at (**a**) 70 °C, (**b**) 110 °C and (**c**, **g**) 150 °C and plasma sintering at 300 W for (**d**) 10min, (**e**) 20 min and (**f**, **h**) 30 min; (**i**) Surface profile of the plasma-sintered silver film.

Figure 4 shows the surface morphologies and chemical composition results of printed silver films with thermal sintering and plasma sintering. It is clearly visible that both treatments promote the coalescence of silver nanoparticles but there are some nuances. In the case of thermal sintering, at 70 °C, the silver nanoparticles are evenly dispersed and loosely connected. Upon sintering at 110 °C, these particles begin to densify, and some of them gather to prepare for diffusing each other. A temperature of 150 °C caused significant neck formation. For plasma sintering, the impact is more pronounced, especially for the silver film produced after 30 minutes. This can probably be attributed to the fast-heating speed and increased thermal energy produced in short time, which allows for a flowing of the structure and the coalescence of the nanoparticles. When plasma sintering silver nanoparticles, the excited high-energy plasma active substances can decompose the organic coating layer covering the outer layer of the nanoparticles and form small molecular compounds through chain scission. These small molecules are volatilized in the low-pressure plasma, leaving behind the uncoated silver nanoparticles, thereby promoting the connection between particles.

EDS analysis was employed to investigate the chemical composition of the silver films from both sintering methods. Three elements, C, O and Ag, were detected in the films, which is in accordance with the original chemical composition of the ink. In terms of silver content, the film formed by thermal sintering at 150 °C for 60 min has a value of 95wt%, which is almost the same as that obtained by plasma sintering at 300 W for 30 min. This indicates the possibility of utilizing a rapid plasma method to produce a conductive silver film. In addition, a plasma-activated ink is also beneficial to the usage of thermally sensitive plastic substrates, broadening options for antennas on flexible substrates.

The above analyses indicate that for the ink conversion, plasma sintering outperformed thermal sintering in terms of electrical performance, sintered film structure and time, as well as without causing damage to the substrates. Considering the PET substrate’s low thermostability and the electrical requirements of the antenna pattern, 300W for 15 minutes were finally chosen for the ink conversion.

### Optimization of antenna parameters

Notch technology can generate a suppression effect in an antenna’s specific frequency band, which is similar to a band-stop filter. It not only solves the problem of interference between systems, but also reduces the demission of the systems. The adoption of three nested "C"-shaped slots is the key to the design of the proposed antenna, which is primarily used to realize the operating bandwidth, achieve three notch performance and decrease the size of metal patch. The influences of parameter values of "C"-shaped slots such as the width (N), the length (W3), the height (L1, L2, L3), and the opening length (W1, W2) on the performance of the antenna were investigated independently. Here, the influence of the width parameter N of the "C"-shaped slot on the notch performance of the antenna was taken as an example. During the optimization procedure, all other antenna parameters are kept constant. When the N value is between 0.2mm and 0.6mm, the ultra-wideband performance of the antenna suffers. Simultaneously, the center frequency of the notch shifts to the left, the coverage width narrows, and the WiMAX band cannot be effectively filtered, and the antenna fails to meet the design requirements. With the gradual increase of the N value to 0.4mm, the S11 curve of the antenna exhibits the best return loss performance. Therefore, we choose 0.4 mm as the optimal N parameter. The process for determining the optimal values of the other parameters is similar, but there is a sequential order, that is, the length and height of the outermost c-slots are determined first, and so on. After a series of simulation optimization, the ultimate antenna size was determined to be: 16 × 17.6 × 0.12 mm^3^, as shown in Table 1.

**Table 1:** Dimensions of the optimized antenna.

Parameter	Value	Parameter	Value	Parameter	Value	Parameter	Value
L	17.6	W4	0.5	W9	6.6	L5	7.8
W	16	W5	0.6	L1	7	L6	0.45
W1	1.2	W6	1	L2	5	H	0.12
W2	0.4	W7	2	L3	3.4	N	0.4
W3	9	W8	2.3	L4	6.6	R	6.5

### Performance of the proposed antenna

**Figure 5:**
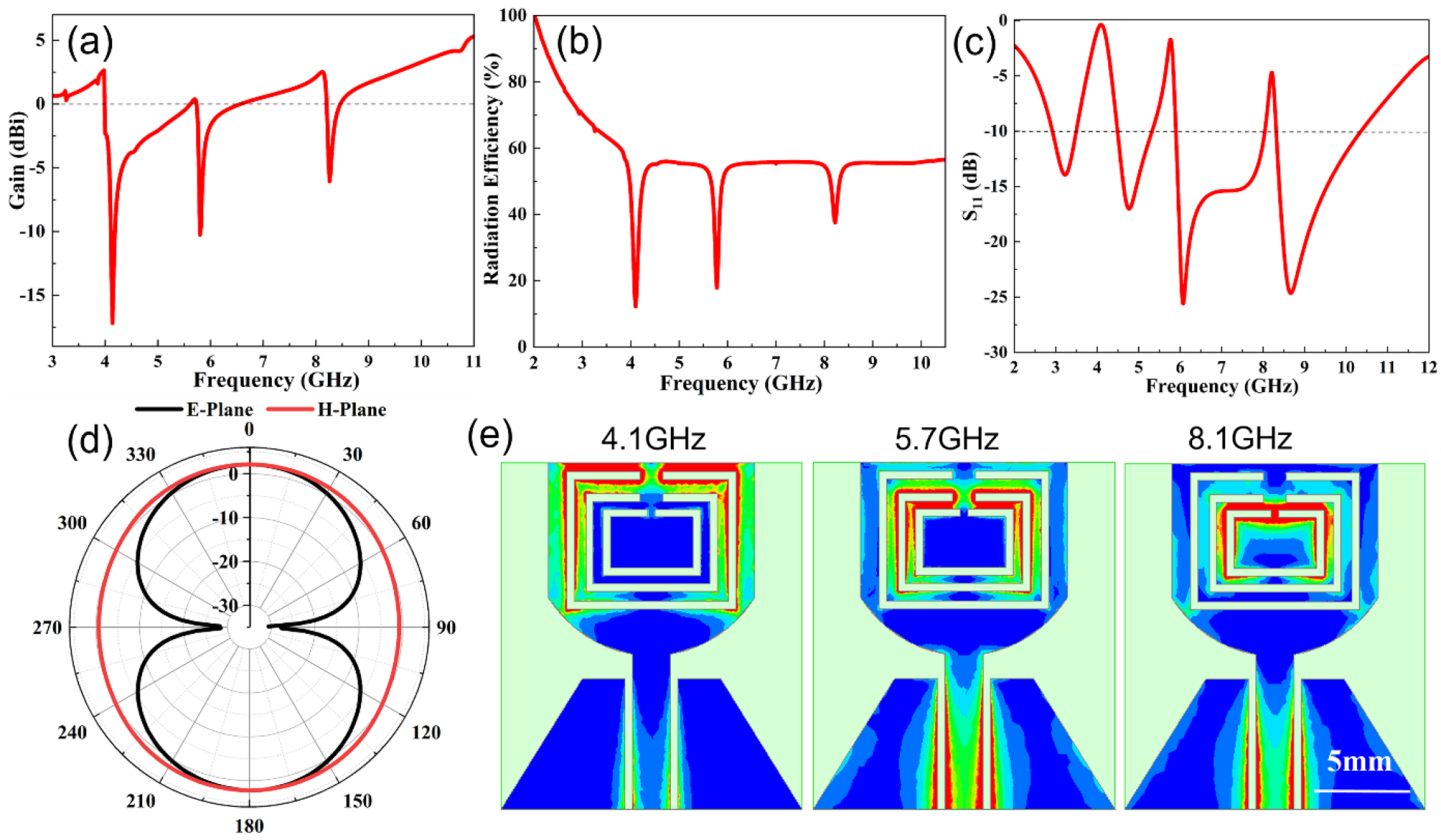
(**a**) Gain, (**b**) radiation efficiency, (**c**) S11 values, (**d**) radiation pattern and (**e**) surface current distribution of the designed flexible trip-notched UWB antenna.

Figure 5a–c shows the simulation results of the antenna in terms of gain, radiation efficiency and S11 values. It can be seen that the antenna operates at 2.9–10.61 GHz, covering the desired UWB frequency band. Meanwhile, the antenna generates notches at 3.51–4.58 GHz, 5.42–5.96 GHz and 8.04–8.31 GHz with S11 values greater than – 10 dB in these areas, successfully shielding the interference from WiMAX, WLAN and X uplink frequency bands. In addition, the smaller return loss values indicate that the antenna has good matching performance and can reduce signal reflection and loss. The gain of the antenna is stable above 2dBi in the whole operating frequency band, with a peak value of 5.5dBi, and the radiation efficiency is stabilized around 60%, which indicates that the antenna has good radiation performance and can meet the application requirements of UWB communication systems. In the WiMAX, WLAN and X notch frequency bands, the gain and radiation efficiency of the antenna are significantly lowered, with minimum gain values of − 17.2 dBi, − 10.2 dBi and – 6 dBi respectively, demonstrating the good notch characteristics of the antenna in theses frequency bands.

The E-plane and H-plane patterns of the antenna at the 6 GHz frequency are given in the Fig. 5d. The E-plane presents an "8"-shaped bidirectional radiation characteristic, while the H-plane shows omnidirectional radiation. This can meet the requirements of a UWB monopole antenna.

The surface current distribution of the antenna was analyzed by HFSS at the notch frequencies of 4.1 GHz, 5.7 GHz and 8.1 GHz to illustrate its notch mechanism, as shown in Fig. 5e. The red and blue color indicates the maximum and the minimum distribution of the current. Obviously, the current is basically concentrated around the three C-shaped slots, indicating the creation of strong notch resonance at these notch frequencies. As can be seen from the first image of Fig. 5e, the flow of surface current on the radiating patch is mainly collected around the outermost "C"-shaped slot structure instead of radiating from the patch edges; consequently, the net radiation of the antenna is blocked at frequency of WiMAX band. Similarly, the middle and the inner "C"-shaped slots show the surface currents that are concentrated around the slot structure, which provides the band-rejection of the WLAN and X-uplink bands. In other words, the energy is focused in the notch structure and not radiated out, thus resulting in good notch characteristics and confirming the rationality of the antenna design.

### Assessment of antenna prototype

**Figure 6:**
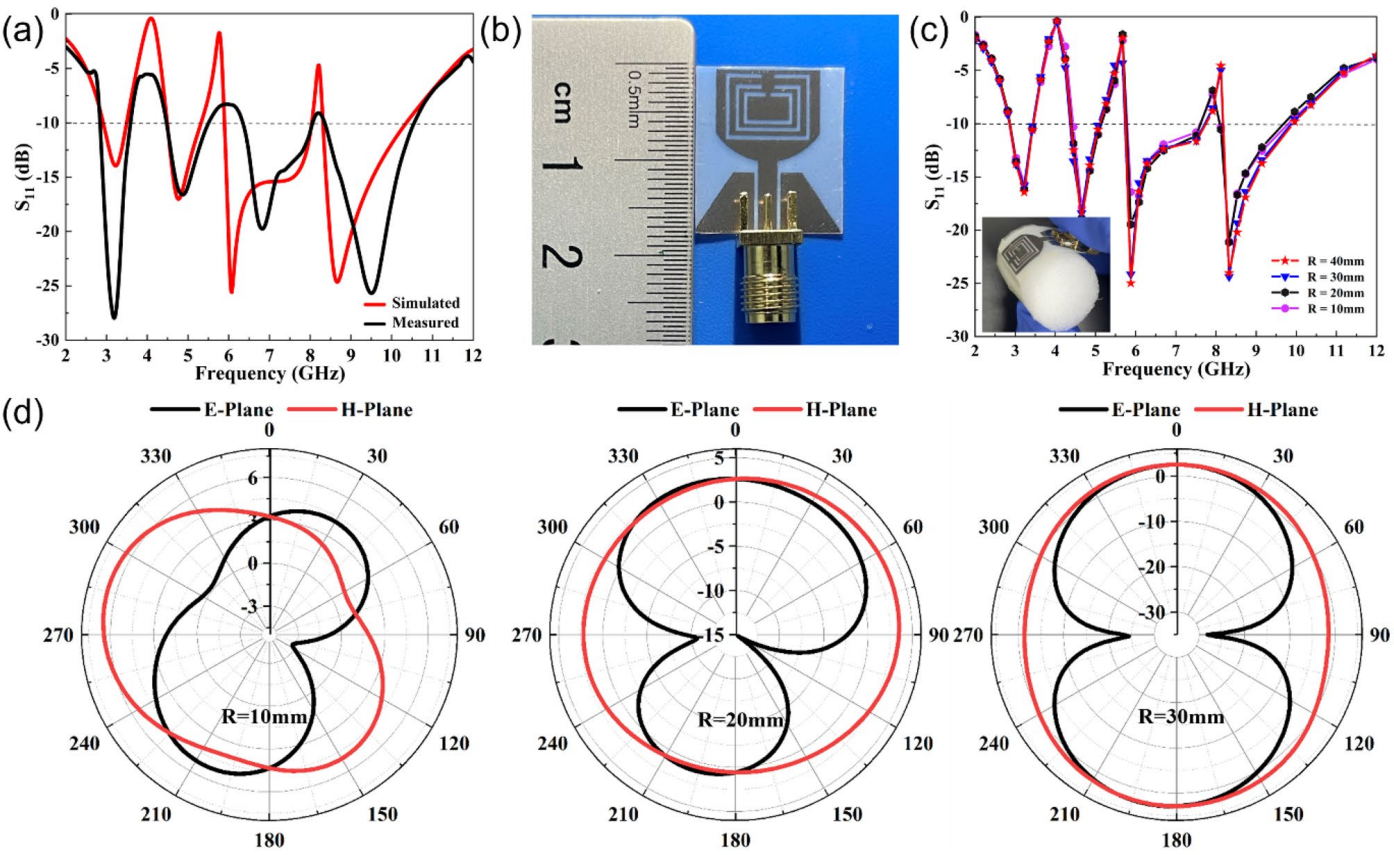
S11 values of the fabricated antenna prototype before (**a**) and after (**c**) bendability test, and (**d**) radiation patterns of the antenna prototype at 6.7GHz when bent along the X-axe with a curvature radius of 10mm, 20mm and 30mm.

Finally, an antenna prototype was fabricated on flexible PET substrates by inkjet printing of the silver nano ink. The S11 values of the prototype were measured using a Keysight E5063A vector network analyzer, and the results are given in the Fig. 6.

As can be seen from Fig. 6a, the changing trends of the measured S11 values are essentially identical with those of the simulated ones in the UWB frequency range. Simultaneously, the antenna prototype successfully suppresses narrow-band interferences from WiMAX, WLAN and X uplink frequency bands, with S11 values larger than-10dB in three frequency bands of 3.5-4.45 GHz, 5.5-6.4 GHz and 8.1-8.3 GHz. The difference between the measured and the simulation results is primarily reflected in the aspects of the right shift of the second notch center frequency and the downward shift or the right shift of some resonance points, which could be related to the errors introduced during the physical processing and the uneven application of conductive silver adhesive connecting the antenna and the RF connector during the measurements.

Figure 6c,d depicts the changes in return loss values and radiation patterns when the antenna prototype is bent along the X-axis with a curvature radius (R) of 10 mm, 20 mm and 30 mm, respectively. With the decrease of R value from 30 mm to 10 mm, the operating frequency range of the antenna varies slightly and the antenna maintains good notch properties, with S11 values less than − 10 dB. The radiation properties are satisfactory when R is 30 mm but distorted when the antenna is bent with a small radius of 10 mm.

From the above analyses, it can be seen that the effects of bending radius on the return loss values of the antenna are not significant but mainly affect the radiation patterns. As for the reason, it might be associated with the impedance matching characteristics of the antenna. Therefore, when designing a bendable antenna, one must consider this issue.

**Figure 7:**
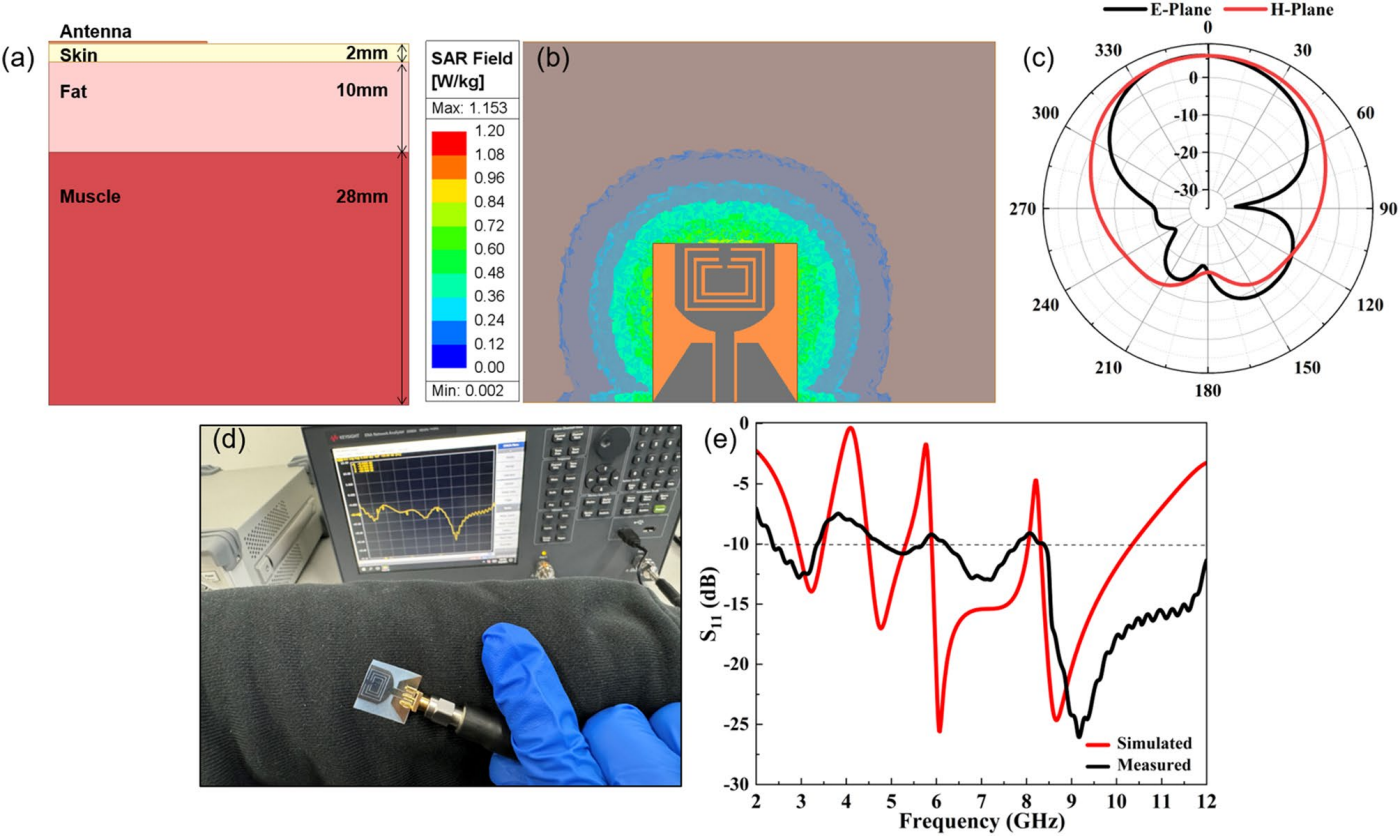
(**a**) The human body phantom utilized for antenna simulation, (**b**,**c**) Simulated SAR values and radiation patterns, (**d**) photo of the antenna prototype placed on the human arm under the measurement and (**e**) the tested S11 values

Besides analyzing the basic parameters, the interaction between human body and antenna was also investigated. A 40 × 60 mm^2^ body phantom consisting of muscle, fat, and skin layers was employed to simulate the antenna's performance on the human body, as shown in Fig. 7a. The required human tissue model parameters were obtained from the reference^70^. The index parameter SAR (specific absorption rate) was used to evaluate the antenna security performance for human body application. According to the EU safety limits, when the maximum SAR value of 10g tissue is less than 2 W/Kg, human health will not be affected by electromagnetic radiation. During the simulation process, the antenna was placed at a height of 3 mm from human tissue to obtain its SAR value and radiation pattern. As shown in Fig. 7b, the proposed antenna has a maximum SAR value of 1.153 W/Kg at an input power of 0.093 W, which is within the safety limit. The radiation pattern results demonstrate that the radiation intensity of the side lobe near the human body is attenuated (Fig. 7c), which might be attributed to the absorption and reflection of electromagnetic waves by human tissues. The simulation results of SAR value and radiation pattern reveal that the influences of the antenna on human health are within an acceptable range and will not affect human health. The antenna prototype is tested on a human arm in free space (Fig. 7d). The measured S11 values (Fig. 7e**)** show that the antenna operates in a range of 2.4 to 12.0 GHz, which deviates somewhat from the simulated values of 2.9 and 10.61GHz, but both cover the desired UWB frequency band with desired triple notch properties. There is a slightly shift at the second notch center frequency, which might be associated with the absorption and reflection of electromagnetic waves by clothes and human tissue. On the whole, the proposed antenna satisfies the design requirements and achieves the notch characteristics in the targeted UWB frequency bands while having a certain of bendability and acceptable SAR values under on-body conditions.

Here, we also compared the created antenna to those reported in the literatures in terms of dimensions, notch numbers, bendability and fabrication technique. As shown in Table 2, the majority of existing flexible UWB antennas were fabricated using an etching procedure, which is not environmentally friendly. Besides, UWB antennas with triple-notch characteristics are relatively large in size and not flexible, making integration on flexible/wearable devices difficult. Compared with the antennas listed in the Table 2, the proposed antenna has three advantages over them. For starters, it can be created on low-cost flexible substrates using a facile, high-efficiency and environmentally friendly ink-based printing process, which has cost and process advantages over those fabricated with traditional etching approach, which needs to go through a series of complex processing steps and produces a large amount of waste liquid, as well as over those fabricated with screen-printing, which needs to make a pattern plate in advance and is unable to modify the antenna pattern in real time. Secondly, it has the smallest size in comparison with others, making it suited for portable UWB system applications, as well as easy integration on flexible and wearable devices. Thirdly, it is bendable and safety for on-body use while maintaining favorable notch characteristics, making it a possible candidate for applications in high-performance UWB systems and flexible/wearable electronic devices. Finally, the utilization of plasma sintering broadens the application range of the antenna substrates and has obvious advantages in terms of time and efficiency in comparison with thermal sintering. A variety of flexible substrate materials, such as polymers, textiles, even paper materials could be possible for the antenna. If the hydrogen is used as the feed gas, the plasma can have a reducing property, which is also beneficial for the use of other metal-based inks such as copper inks. Of course, there are some other flexible UWB antennas with notch characteristics. Although these antennas have achieved structural flexibility, they are created via non-inkjet printing process. Kapton film, polyimide, is often used as a substrate for flexible electronics. It was not chosen as a substrate in this work because it is less compatible with the purchased silver ink than PET, and an additional hydrophilic surface treatment process is required for it during inkjet printing to ensure the precision of the printed antenna pattern in geometry. In addition, polyimide is almost opaque and has a wide thermal stability, and we aim to explore the antenna applications on transparent and temperature-sensitive flexible substrates and highlight the advantages of plasma sintering.

**Table 2:** Comparison of the proposed antenna with others reported in the literatures.

Ref.	Dimensions (L×W×H, mm^3^)	Operating frequency band (GHz)	Notch number	Notch frequency band (GHz)	Notch technique	Bendability	Fabrication approach	Metallization method	Substrate
^11^	42.5×30×0.6	3.25–13	Single	5.7–6.2	SRR	Yes	Etching	/	Teflon
^16^	40×30×1.5	3.1–10.6	Single	5.15–5.875	Slots	Yes	Not given	/	Compressed Neoprene
^17^	30×20×0.2	2.76–10.6	Dual	3.3–3.6, 5.15–5.85	U-shaped slot	Yes	Not given	/	Polyimide
^21^	45×35×0.6	1.20–13	Triple	2.0–2.7, 3.45–3.80, 5.15–6.20	SRRs and DGS	Yes	Etching	/	Teflon
^22^	32×25×0.064	2.8–5.35	Triple	2.35–2.5, 3.18–3.82, 4.15–5.42	SRRs	Yes	Etching	/	N/A
^23^	27×20×0.8	2.8–11.6	Dual	3.29–3.68, 5.1–6.1	CSRR	No	Etching	/	FR4
^28^	40×29×1.6	2.70–11.06	Triple	3.22–3.83, 4.49–5.05, 7.49–8.02	C slot, resonator and parasitic stub	No	Etching	/	FR4
^29^	62×60×0.125	2.05–14	Dual	2.4–3.7, 5.15–5.725	DGS resonator slot	Yes	Air-Brush-printing	Thermal but not given detailed parameters	Kapton Polyimide
^30^	55×40×0.125	1.77– 6.95	N/A	N/A	DGS	Yes	Screen printing	150 °C for 60min	Kapton
^31^	49×34×0.05	3.1–10.6	Single	5.15–5.825	DGS	Yes	Etching	/	PET
^32^	34.9×31.3×1.6	2.9–10.5	Quad	2.53–3.15, 3.23–3.68, 3.92–4.30, 5.49–6.19	EBG-Unit cell	No	Etching	/	FR4
^33^	66.3×66.3×0.813	3.1–10.6	Dual	3.6–3.9, 5.6–5.8	Mushroom EBG	No	Etching	/	Rogers RO4003C
^34^	52×40×0.762	2.7–11.7	Dual	3.45–3.9, 6.7–7.65	Spiral EBG	No	Etching	/	N/A
^36^	38×23×0.6	2.33–11.3	Dual	4.4–4.99, 5.15–5.85	A circular slotted rectangular section on feed line	Yes	Not given but using copper foil	/	Jeans
Our previous work^3^	27×38×0.12	1.9–10.75	Triple	3.2–3.8, 5.3–6.2, 7.8–8.5	“U” and “C”-like slots	Yes	Inkjet printing	Thermal Sintering,130°C for 60min	PET
This work	17.6×16×0.12	2.9–10.61	Triple	3.5–4.45, 5.5–6.4, 8.1–8.3	C slots	Yes	Inkjet printing	Plasma Sintering, 300W for 15min	PET

Overall, the usage of inkjet printing, flexible substrates and low temperature-activated conductive ink has provided a facile method for the fabrication of flexible/wearable UWB antennas. However, it concurrently brings challenges to the materials, printing process and antenna structure design. Future research will focus on innovative ideas in material science, process technology, creative engineering solutions in mechanical and electromagnetic designs, and the intelligent combination of these parts.

## Conclusions

A miniaturized tri-notched flexible UWB Antenna is developed using an inkjet-printable and plasma-activated silver nano ink in a simple and facile additive procedure. The ink shows good printability, and can produce highly conductive patterns on flexible PET substrates with an efficient plasma sintering technique. The developed antenna has a simple structure and a compact size of 17.6 mm × 16 mm × 0.12 mm. It operates at the targeted UWB frequency band and generates notches at 3.51–4.58 GHz, 5.42–5.96 GHz and 8.04–8.31 GHz, successfully shielding the interference from WiMAX, WLAN and X uplink frequency bands. The notch properties are achieved by introducing three nested "C" slots of different sizes on the radiating patch of the antenna. Both simulation and test results demonstrate that the antenna can produce signal radiation in the targeted UWB frequency band while generating the desired notch properties and having acceptable SAR values on-body, making it a good candidate for usage in flexible or wearable wireless electronic devices.

## Data Availability

The authors declare that the data supporting the findings of this study are available within the paper.
